# Integrating Network Pharmacology and Pharmacological Evaluation for Deciphering the Action Mechanism of Herbal Formula Zuojin Pill in Suppressing Hepatocellular Carcinoma

**DOI:** 10.3389/fphar.2019.01185

**Published:** 2019-10-09

**Authors:** Wei Guo, Jihan Huang, Ning Wang, Hor-Yue Tan, Fan Cheung, Feiyu Chen, Yibin Feng

**Affiliations:** ^1^School of Chinese Medicine, Li Ka Shing Faculty of Medicine, The University of Hong Kong, Hong Kong, China; ^2^Center for Drug Clinical Research, Shanghai University of Traditional Chinese Medicine, Shanghai, China

**Keywords:** Zuojin pill, hepatocellular carcinoma, network pharmacology, pharmacological evaluation, cell proliferation and survival

## Abstract

Hepatocellular carcinoma (HCC) is a kind of complicated disease with an increasing incidence all over the world. A classic Chinese medicine formula, Zuojin pill (ZJP), was shown to exert therapeutic effects on HCC. However, its chemical and pharmacological profiles remain to be elucidated. In the current study, network pharmacology approach was applied to characterize the action mechanism of ZJP on HCC. All compounds were obtained from the corresponding databases, and active compounds were selected according to their oral bioavailability and drug-likeness index. The potential proteins of ZJP were obtained from the traditional Chinese medicine systems pharmacology (TCMSP) database and the traditional Chinese medicine integrated database (TCMID), whereas the potential genes of HCC were obtained from OncoDB.HCC and Liverome databases. The potential pathways related to genes were determined by gene ontology (GO) and pathway enrichment analyses. The compound-target and target-pathway networks were constructed. Subsequently, the potential underlying action mechanisms of ZJP on HCC predicted by the network pharmacology analyses were experimentally validated in HCC cellular and orthotopic HCC implantation murine models. A total of 224 components in ZJP were obtained, among which, 42 were chosen as bioactive components. The compound-target network included 32 compounds and 86 targets, whereas the target-pathway network included 70 proteins and 75 pathways. The *in vitro* and *in vivo* experiments validated that ZJP exhibited its prominent therapeutic effects on HCC mainly *via* the regulation of cell proliferation and survival though the EGFR/MAPK, PI3K/NF-κB, and CCND1 signaling pathways. In conclusion, our study suggested combination of network pharmacology prediction with experimental validation may offer a useful tool to characterize the molecular mechanism of traditional Chinese medicine (TCM) ZJP on HCC.

## Introduction

As the third predominant cause of cancer-related death in the world, hepatocellular carcinoma (HCC) is a kind of complicated disease with various risk factors, such as hepatitis B viral or hepatitis C viral infection, obesity, and alcohol abuse (Ferlay et al., 2015). The incidence of HCC is mounting all over the world, whereas the prognosis of this disease is still far from satisfactory (Sherman, 2008). There are five commonly used treatments for HCC currently, namely, liver transplantation, transcatheter arterial chemoembolization, surgical resection, radiofrequency ablation, and sorafenib (Llovet et al., 2015). However, as most HCC patients are diagnosed at middle or later disease stages, only sorafenib treatment is still feasible for these patients. What is worse, fewer than 20% patients are able to respond to sorafenib, whereas moderate or severe side effects are frequently caused by sorafenib. There is an essential need to develop more effective and less toxic therapies for HCC (Zhu et al., 2016). Traditional Chinese medicine (TCM) has been used clinically in Asia for more than 2,000 years. As one of the most popular complementary and alternative medicine modalities in China, TCM has been gradually accepted by non-Chinese due to its prominent efficacy, rich resource, and less toxicity. There have been many TCM formulas used alone or as an adjuvant to conventional chemotherapy in the clinical treatment of cancers (Kim et al., 2012; Xu et al., 2012a; Gavaraskar et al., 2015).

Zuojin pill (ZJP) is a drug pair commonly used in TCM. It consists of only two herbs, namely, Coptidis Rhizoma (CR, Huang-lian in Chinese) and Evodiae Fructus (EF, Wu-chu-yu in Chinese). The ratio of CR and EF is 6:1 (w/w). CR is obtained from the dried rhizome of *Coptis chinensis* Franch and widely applied for the treatment of various diseases, such as gastrointestinal disorders, hepatic damages, and diabetes (Wang et al., 2015a; Lam et al., 2016). EF is obtained from the immature fruit of *Evodia rutaecarpa* Benth and widely applied for the treatment of headache, inflammation, and hypertension (Xu et al., 2012b). Alkaloids are proved to be the primary compounds of both CR and EF. Previous studies revealed that ZJP, its comprising herbs CR and EF, as well as the active compounds exhibited multiple pharmacological effects against cancer *via* various mechanisms of action (Wang et al., 2009; Wang et al., 2010a; Chou et al., 2017; Pan et al., 2017). ZJP extracts exerted its anticancer activity on colorectal cancer cells through the attenuation of the 5-HTR1D-Wnt/β-catenin signaling pathway (Pan et al., 2017). ZJP showed anticancer activity against sarcoma cancer *via* its effects on gene expression and activities of serum tumor markers (Wang et al., 2009). Notably, ZJP markedly inhibited tumor growth in orthotopic HepG2 xenograft-bearing immunocompetent mice model (Chou et al., 2017). However, the chemical and pharmacological foundations of ZJP in inhibiting human cancers, especially HCC, was not globally evaluated with appropriate approaches.

TCM is a complex system with multiple targets and synergistic or antagonistic interactions among its components (Ma et al., 2015). Unlike western medicine of “one target, one drug,” the concept of the integrity of the whole human body is emphasized in the theory of TCM. Because of its complexity in composition, conventional pharmacological approaches to experimentally identify the unique action of mechanism may not be applicable to TCM research. Along with the rapid development of bioinformatics, the newly emerging network pharmacology is based on big databases and has become a useful tool to characterize the action mechanisms of complicated drug system in detail, from the molecular level to the pathway level (Chen et al., 2016). Network pharmacology meets the key ideas of the holistic philosophy of TCM (Li and Zhang, 2013). As a state-of-the-art technique, this method updates the research paradigm from the current “one target, one drug” mode to a new “network target, multicomponents” mode. It helps to evaluate the rationality and compatibility of TCM by providing the detailed compound-target and target-pathway networks. It has been widely applied in the mechanism study of TCM for the treatment of complex diseases, such as cancer, asthma, and cardiovascular disorders. Successful attempts to apply this method to investigate complex TCM have been achieved in our laboratory (Hong et al., 2017a; Hong et al., 2017b; Huang et al., 2017a; Huang et al., 2017b; Huang et al., 2017c) and other researchers (Liang et al., 2014; Chen et al., 2018; Wang et al., 2018; Zheng et al., 2018; Zuo et al., 2018).

**Figure 1:**
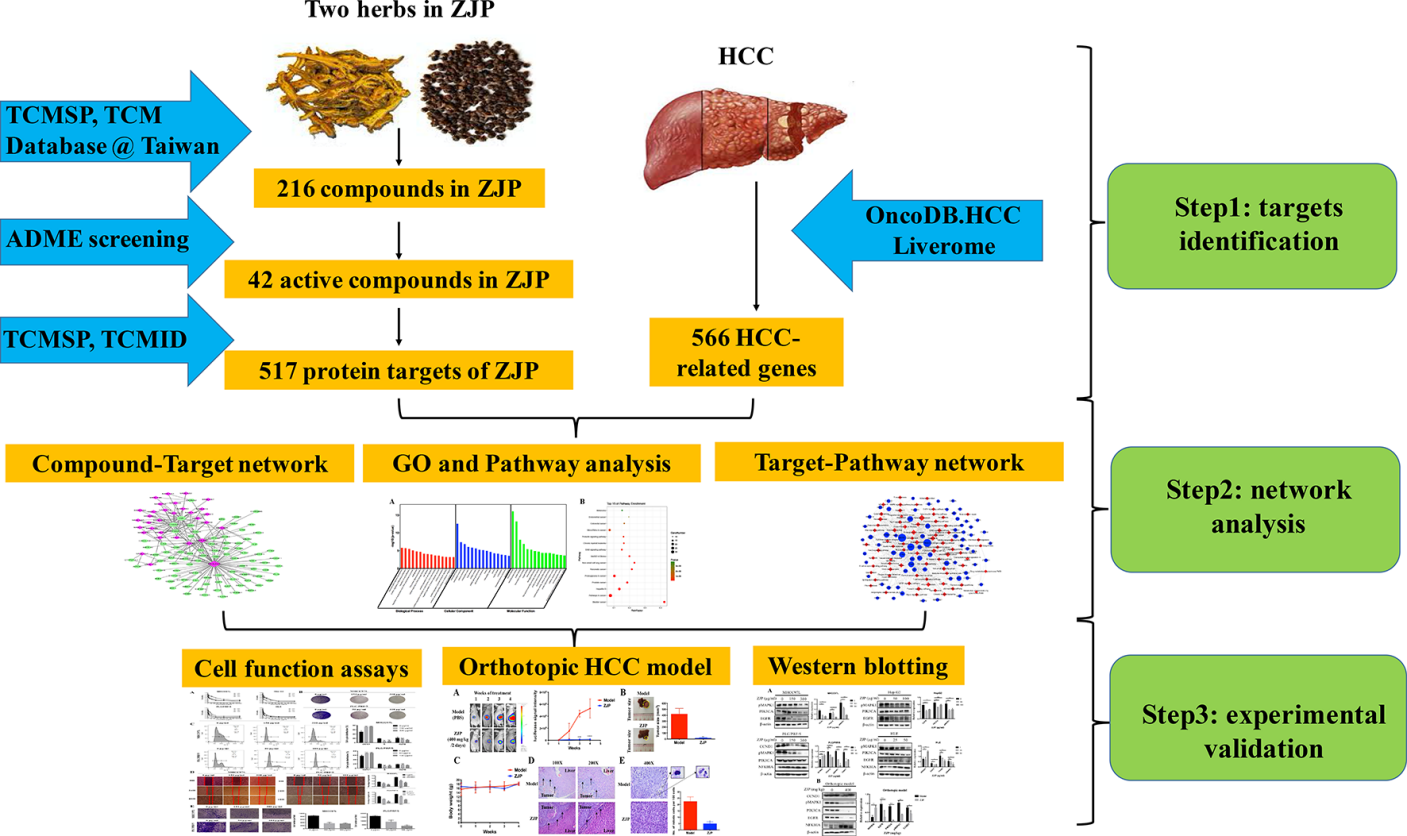
The technical strategy of the current study.

In the current study, we used computational tools and resources to investigate the pharmacological network of ZJP on HCC to predict the active compounds and potential protein targets and pathways. In addition, *in vitro* and *in vivo* experiments were also conducted to validate the potential underlying mechanism of ZJP on HCC, as predicted by network pharmacology approach. The detailed technical strategy of the current study was shown in **Figure 1**.

## Materials and Methods

### Network Pharmacology-Based Analysis

#### Identification of Candidate Components in ZJP

All components of the two Chinese medicinal herbs in ZJP (Huang-lian and Wu-chu-yu) were retrieved from the traditional Chinese medicine systems pharmacology (TCMSP) database (http://tcmspw.com/) (Ru et al., 2014).

#### Screening Strategy for Bioactive Components in ZJP

The oral TCM must overcome the barriers posed by absorption, distribution, metabolism, and excretion (ADME) processes to be active. In ADME processes, oral bioavailability (OB) is one of the most significant pharmacokinetic parameters (Xu et al., 2012c). High OB is usually an essential indicator to determine the drug-likeness (DL) index of active substances. The substances with OB ≥ 30% were regarded to have high OB.

As a qualitative concept applied in drug design to estimate the druggability of a molecule (Tao et al., 2013), the DL index is useful for rapid screening of active substances. In the DrugBank database, the average DL index is 0.18. The substances with DL index ≥0.18 were regarded to have high druggability.

Therefore, the compounds in ZJP with OB ≥ 30% and DL index ≥0.18 were selected as active substances in the current study.

#### Prediction of Drug Targets for ZJP

The protein targets of the active substances in ZJP were retrieved from the TCMSP database and the traditional Chinese medicine integrated database (TCMID, http://www.megabionet.org/tcmid/).

#### Collection of Gene Targets for HCC

HCC-related human genes were collected from two databases, namely, OncoDB.HCC (http://oncodb.hcc.ibms.sinica.edu.tw/index.htm) (Su et al., 2007) and Liverome (http://liverome.kobic.re.kr/index.php) (Lee et al., 2011). Then, the protein targets of ZJP were mapped with HCC using the therapeutic target database (TTD, http://bidd.nus.edu.sg/group/cjttd/TTD_HOME.asp), the comparative toxicogenomics database (CTD, http://ctdbase.org/), and PharmGKB (https://www.pharmgkb.org/).

#### Gene Ontology and Pathway Enrichment Analysis for HCC-Related Targets of ZJP

The gene ontology (GO) and pathway enrichment analyses were conducted using the functional annotation tool of DAVID Bioinformatics Resources 6.7 (http://david.abcc.ncifcrf.gov/) (Huang da et al., 2009). Terms with thresholds of Count ≥ 2 and Expression Analysis Systematic Explorer (EASE) scores ≤ 0.05 were chosen in functional annotation clustering.

#### Construction of Networks and Analysis

To further characterize the molecular mechanism of ZJP on HCC, the compound-target and target-pathway networks were generated using Cytoscape 3.3.0 (Smoot et al., 2011). In these graphical networks, the compounds, proteins, or pathways were expressed as nodes, whereas the compound-target or target-pathway interactions were expressed as edges.

### Experimental Validation

#### Preparation of ZJP Aqueous Extract

Coptidis Rhizoma (voucher No. 44-1) and Evodiae Fructus (voucher No. 0018) were obtained and authenticated by the executive manager of dispensary of School of Chinese Medicine, The University of Hong Kong under the guidance of Chinese Pharmacopeia 2015 edition. The herbs were vouched and stored in specimen room of School of Chinese Medicine, The University of Hong Kong. To prepare the aqueous extract of ZJP, 60 g Coptidis Rhizoma and 10 g Evodiae Fructus were soaked in 700 ml distilled water for 30 min, and then were decocted for 1 h. The solvent was centrifuged at 10,000 rpm for 30 min, and the supernatant was collected. This extraction step was repeated twice, and the supernatants were merged and then evaporated to dryness. The dried powder was redissolved in distilled water to 10 mg/ml and filtered with a 0.22 μm pore-size filter and stored at -20°C for further use.

#### Phytochemical Analysis of ZJP

Fingerprinting analysis was conducted by UPLC to identify the chemical profile of ZJP. In detail, 5 μl of ZJP aqueous extract (1 mg/ml) and standards were respectively injected into the UPLC system (Thermo Fisher Scientific, USA) and separated on C18 ODS column (250×4.6 mm id, ACE, Scotland) with gradient elution. 0.085% H_3_PO_4_ (A) and acetonitrile (B) was used as mobile phase and the gradient elution procedure was as follows: 0 min, A:B = 97:3; 3 min, A:B = 97:3; 16 min, A:B = 80:20; 50 min, A:B = 76:24; 55 min, A:B = 65:35; 65 min, A:B = 50:50; and 70 min, A:B = 35:75. The flow rate was 1.0 ml/min and the detection was under the absorption wavelength of 280 nm.

#### Cell Culture

Human HCC HepG2, MHCC97L, PLC/PRF/5, and HLE cells were chosen for the following experiments. HepG2 and PLC/PRF/5 cells were commercially obtained from American Type Culture Collection (ATCC; Manassa, VA, USA); HLE cells were purchased from JCRB (Japan); and MHCC97L cells were kindly gifted by Professor Man Kwan from Department of Surgery, The University of Hong Kong. Cells were cultured in DMEM medium supplemented with 10% FBS, 100 U/ml penicillin, and 100 mg/ml streptomycin and maintained at 37°C in a humidified chamber with 5% CO_2_.

#### Cell Viability Assay

HCC cells (5,000 cells/well) were seeded in 96-well plates and incubated for 24 h. After pretreatment with different concentrations of ZJP (0, 7.8125, 15.625, 31.25, 62.5, 125, 250, 500, and 1,000 μg/ml) for 24, 48, and 72h, 10 μl of 3-(4,5-Dimethylthiazol-2-yl)-2,5-diphenyltetrazolium bromide solution (MTT, 5 mg/ml; Sigma, USA) was added to each well and then cells were cultured at 37°C for another 4 h. Then, the supernatants were discarded and 100 μl of DMSO was added to each well. The absorbance was measured at 595 nm using Multiskan MS microplate reader (Labsystems, Finland).

#### Colony Formation Assay

MHCC97L and PLC/PRF/5 cells were seeded in 6-well plates at 5000 cells per well and incubated for 24 h. Then, cells were treated with or without ZJP (150 and 300 μg/ml for MHCC97L, 50 and 100 μg/ml for PLC/PRF/5) for continuous 10 days. After fixation with 4% paraformaldehyde for 30 min, the cells were stained with crystal violet solution for 2 h. The photographs of the colonies were taken manually after washed with water.

#### Flow Cytometry for Cell Cycle Analysis

MHCC97L and PLC/PRF/5 cells (5 × 10^5^ cells/well) were seeded in 6-well plates. After incubation for 12 h, the medium was changed to serum-free DMEM for another 12 h and then cells were treated with or without ZJP (150 and 300 μg/ml for MHCC97L, 50 and 100 μg/ml for PLC/PRF/5) for 24 h. Cells were collected and fixed with 70% ethanol at 4°C overnight. After fixation, the cells were stained with propidium iodide (PI, 50 μg/ml, Sigma-aldrich, USA) for 45 min in dark. The cell samples were tested with Canto II flow cytometer (BD Bioscience, USA) for cell cycle analysis.

#### Wound-Healing and Transwell Invasion Assay

For wound-healing assay, MHCC97L and PLC/PRF/5 cells were incubated in 6-well plates with 100% confluence. A denuded area was scrapped using a plastic pipette tip on the cell monolayer. Medium was removed and the monolayer was washed 3 times with PBS. Then, medium containing different concentrations of ZJP (150 and 300 μg/ml for MHCC97L, 50 and 100 μg/ml for PLC/PRF/5) was added to each well and cell movements into the wound area were obtained after 0, 24, and 48 h incubation with a microscope. For transwell invasion assay, millicell cell culture inserts in 24-well plates were pretreated with 100 μl of cold Matrigel (BD Biosciences, USA, diluted 1:4 with cold PBS) for 2 h at 37°C. MHCC97L and PLC/PRF/5 cells (1 × 10^5^ cells/well) were seeded to the chamber with 200 μl of serum-free DMEM and then incubated with or without ZJP (150 and 300 μg/ml for MHCC97L, 50 and 100 μg/ml for PLC/PRF/5) at 37°C for 24 h. The invaded cells are fixed with 4% paraformaldehyde for 30 min and stained with crystal violet solution for 2 h and then counted with a light microscope.

#### Orthotopic HCC Implantation Murine Model

All animals received human care throughout the experiments and the study protocols were approved by the Committee on the Use of Live Animals in Teaching and Research (CULATR). In brief, 5×10^6^ luciferase-tagged MHCC97L cells were subcutaneously injected into the left waist of 5-week-old male BALB/c nu/nu athymic nude mice to establish xenografted tumor. When the subcutaneous tumor reached 1 cm in diameter, it was dissected and cut into small cubes (approximately 1 mm^3^). The small tumor cube was orthotopically implanted into the left liver lobe of 5-week-old male BALB/c nu/nu athymic nude mice to establish orthotopic HCC implantation murine model. One week after implantation, the growth of liver tumor was checked under *in vivo* live imaging system (IVIS Spectrum, Perkin-Elmer, USA) by injecting luciferin (i.p., 150 mg/kg) into the mice. All tumor-presenting mice were then randomized into model and ZJP treatment groups (n = 5) receiving gavage of PBS and ZJP (400 mg powder/kg/2 day) respectively for 4 weeks. Liver tumor growth was monitored weekly. At the end of treatment, the nude mice were humanely sacrificed to collect tissues.

#### Western Blotting

For cell samples, HCC cells (5 × 10^5^ cells/well) were seeded in 6-well plates. After incubation overnight, the cells were treated with or without ZJP for 24 h (50 and 100 μg/ml for HepG2 and PLC/PRF/5, 150 and 300 μg/ml for MHCC97L, 25 and 50 μg/ml for HLE). The cells were harvested using a microscraper (Corning). For tissue samples, the tumor tissues were collected after 4 weeks’ treatment of ZJP. The expression levels of PIK3CA, EGFR, NFKIBA, CCND1, and pMAPK1 were examined by western blotting. In brief, the whole-cell extracts and tumor homogenates were lysed with RIPA buffer supplemented with proteinase inhibitor (1% PMSF, 0.5% aprotinin, and 0.5% leupeptin) and phosphatase inhibitor (1 mM Na_3_VO_4_ and 1 mM NaF) on ice for 30 min. Subsequently, the lysates were centrifuged at 14,000 rpm for 10 min at 4ºC. The protein concentration was detected using bovine serum albumin (BSA; Sigma, MO, USA) as a standard. Equal amount of protein in each sample was resolved by sodium dodecyl sulfate-polyacrylamide gel electrophoresis (SDS-PAGE) and transferred onto a polyvinylidene fluoride membrane (PVDF; Biorad, USA). Next, the membrane was blocked with 5% BSA in Tris buffered saline-Tween 20 (TBST) buffer (10 mmol/L Tris, 150 mmol/L NaCl, 1% Tween 20, pH 7.4) for 2 h at room temperature. The blots were then incubated with primary antibodies (anti-PIK3CA, anti-EGFR, anti-NFKIBA, anti-CCND1, and anti-pMAPK1 antibody at 1:1000; Abcam, UK) at 4ºC overnight. After washed with TBST buffer for three times, the blots were incubated with the secondary antibody (Abcam, UK) for 2 h at room temperature. Immunoreactivity was determined using an advanced ECL kit (GE Healthcare, UK) and visualized using a chemiluminescence imaging system (Biorad).

#### Histopathological Examination

Livers from the orthotopic implanted mice were dissected and fixed in 4% formalin buffer. Paraffin-embedded blocks were prepared and sections at 5 μm thickness were cut and stained with hematoxylin and eosin for histological examination.

#### Statistical Analysis

Statistical analysis was processed with Prism 6 software. Data were expressed as the mean ± SD and analyzed using Student’s t-test. Differences between groups were considered to be statistically significant if values of P < 0.05.

## Results

### Network Pharmacology-Based Analysis

#### Identification of Bioactive Components in ZJP

The fingerprinting analysis of ZJP aqueous extract were conducted by the UPLC. As shown in **Supplement 1**, approximately 14 chromatographic peaks were identified as the phytochemical profile of ZJP. Among them, two compounds of main peaks of UPLC chromatogram were identified as berberine and palmatine based on their retention time and UV spectrum.

A total of 224 components in ZJP were obtained from TCMSP database, 48 of which belong to CR and 176 to EF (**Supplement 2**). Of note, there were eight common components shared in these two herbs, namely, berberine, isovanillin, heriguard, obacunoic acid, obamegine, obacunone, limonin, and quercetin. Among the 48 components in Coptidis Rhizoma, 26 (54.2%) met the requirement of OB ≥30% and 14 (29.2%) met the requirements of OB ≥30% and DL index ≥0.18. Among the 176 components in Evodiae Fructus, 106 (60.2%) met the requirement of OB ≥30% and 31 (29.2%) met the requirements of OB ≥30% and DL index ≥0.18 (**Table 1**). After eliminating the overlaps, 42 components were chosen as candidate bioactive components for further analyses and the detailed information was shown in **Table 2**.

**Table 1:** Number of components in ZJP with OB ≥ 30% and DL index ≥ 0.18.

Herbs	Total	OB ≥ 30%	OB ≥ 30% and DL ≥ 0.18
Coptidis Rhizoma	48	26 (54.2)	14 (29.2)
Evodiae Fructus	176	106 (60.2)	31 (17.6)

**Table 2:** Information for candidate bioactive components of ZJP.

Number	Molecule Name	OB (%)	DL	Molecules structure	Herb
MOL001	Berberine	36.86	0.78	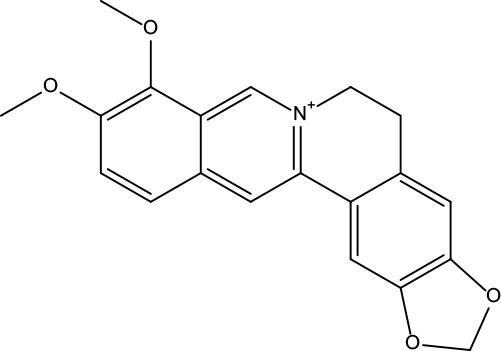	Coptidis Rhizoma/Evodiae Fructus
MOL011	Obacunone	43.29	0.77	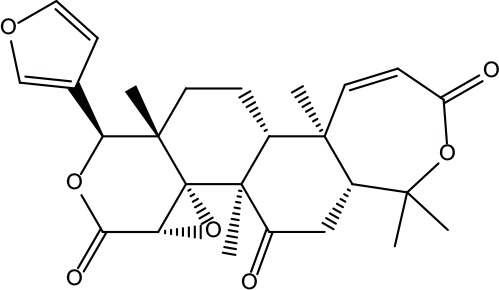	Coptidis Rhizoma/Evodiae Fructus
MOL013	Berberrubine	35.74	0.73	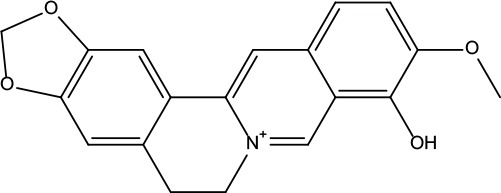	Coptidis Rhizoma
MOL016	Epiberberine	43.09	0.78	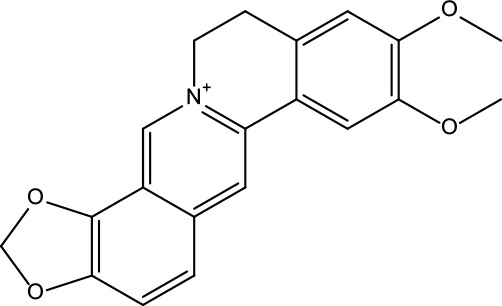	Coptidis Rhizoma
MOL022	(R)-Canadine	55.37	0.77	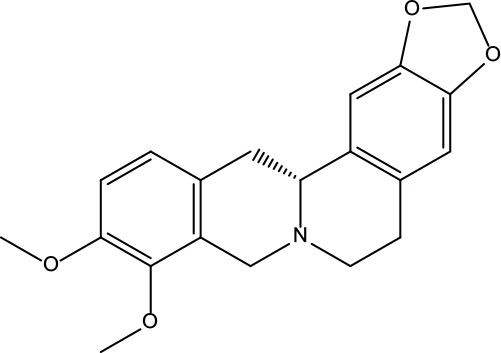	Coptidis Rhizoma
MOL023	Berlambine	36.68	0.82	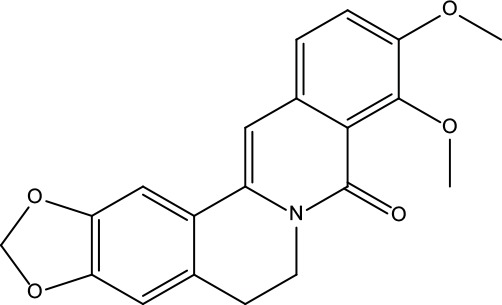	Coptidis Rhizoma
MOL026	Corchoroside A_qt	104.95	0.78	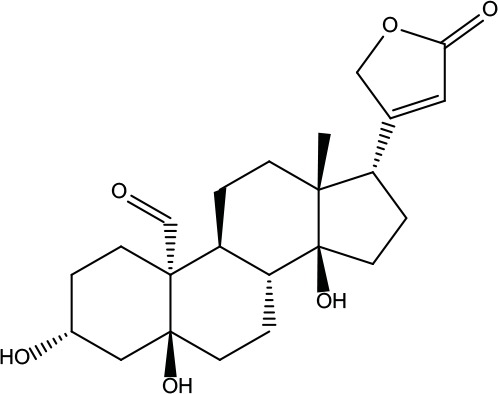	Coptidis Rhizoma
MOL028	Magnograndiolide	63.71	0.19	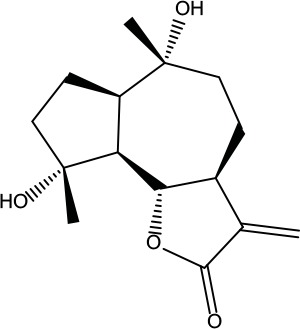	Coptidis Rhizoma
MOL029	Palmidin A	35.36	0.65	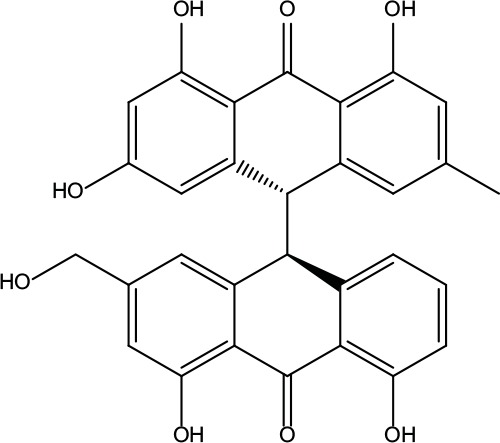	Coptidis Rhizoma
MOL032	Palmatine	64.6	0.65	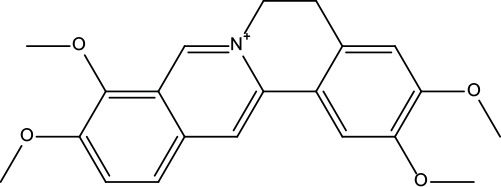	Coptidis Rhizoma
MOL034	Quercetin	46.43	0.28	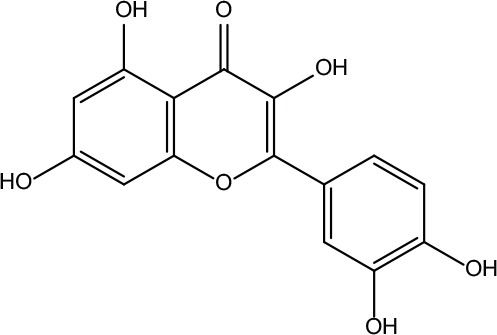	Coptidis Rhizoma/Evodiae Fructus
MOL038	Coptisine	30.67	0.86	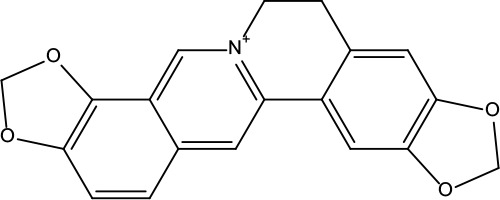	Coptidis Rhizoma
MOL041	Worenine	45.83	0.87	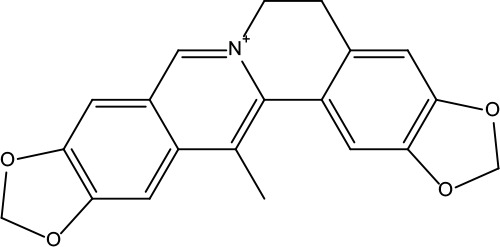	Coptidis Rhizoma
MOL047	Moupinamide	86.71	0.26	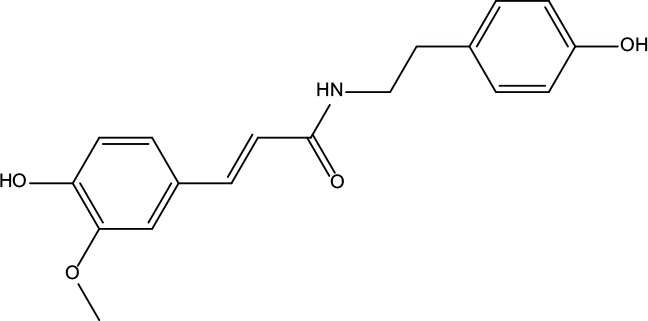	Coptidis Rhizoma
MOL095	Rutaecarpine	40.3	0.6	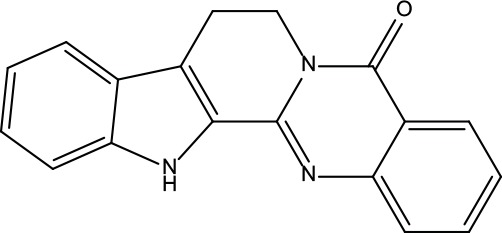	Evodiae Fructus
MOL104	Isorhamnetin	49.6	0.31	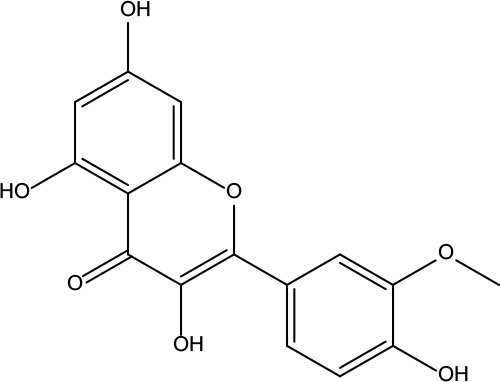	Evodiae Fructus
MOL106	Beta-sitosterol	36.91	0.75	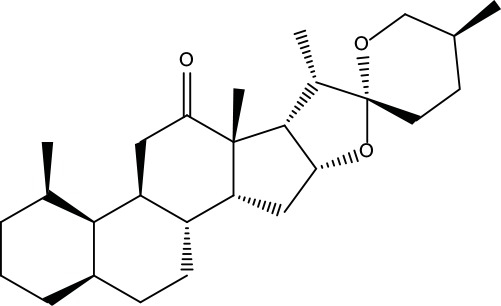	Evodiae Fructus
MOL107	Sitosterol	36.91	0.75	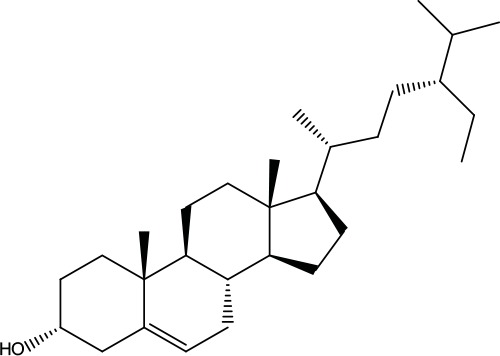	Evodiae Fructus
MOL117	Rutaevine	66.05	0.58	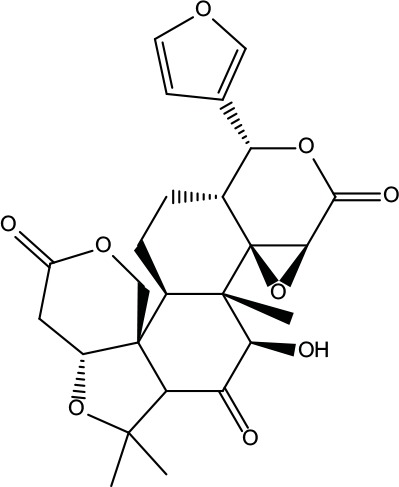	Evodiae Fructus
MOL118	Rutalinidine	40.89	0.22	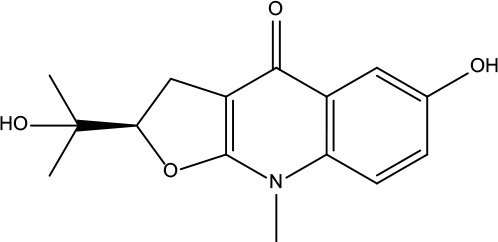	Evodiae Fructus
MOL122	1-Methyl-2-[(Z)-pentadec-10-enyl]-4-quinolone	48.45	0.46	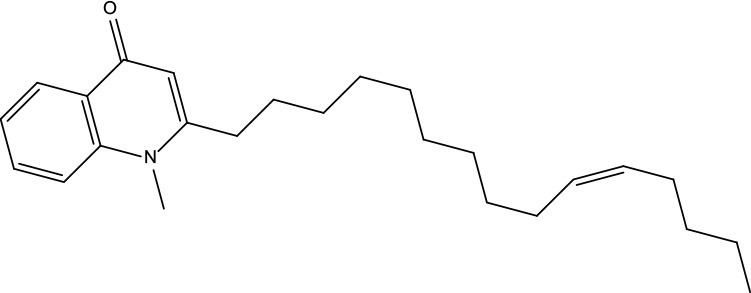	Evodiae Fructus
MOL125	1-Methyl-2-[(Z)-undec-6-enyl]-4-quinolone	48.48	0.27	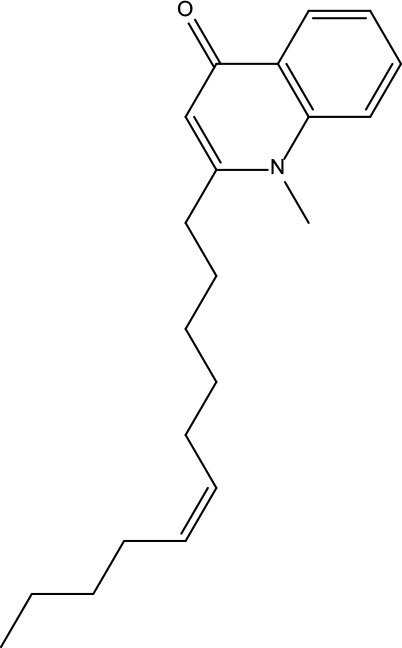	Evodiae Fructus
MOL131	Dihydrorutaecarpine	42.27	0.6	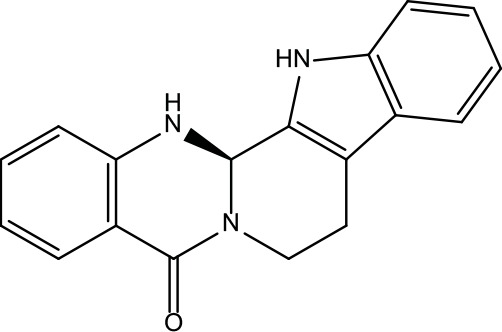	Evodiae Fructus
MOL132	1-Methyl-2-pentadecyl-4-quinolone	44.52	0.46	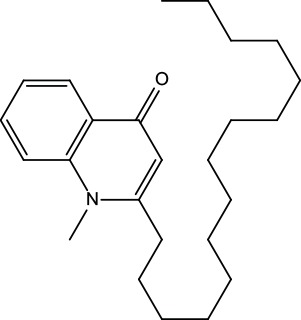	Evodiae Fructus
MOL133	Evodiamine	86.02	0.64	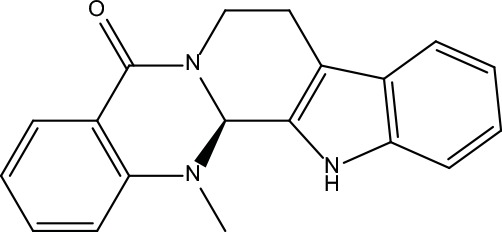	Evodiae Fructus
MOL134	1-(5,7,8-Trimethoxy-2,2-dimethylchromen-6-yl)ethanone	30.39	0.18	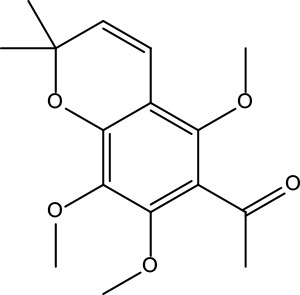	Evodiae Fructus
MOL137	Hydroxyevodiamine	72.11	0.71	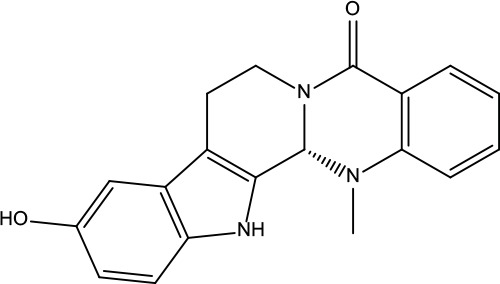	Evodiae Fructus
MOL138	1-Methyl-2-undecyl-4-quinolone	47.59	0.27	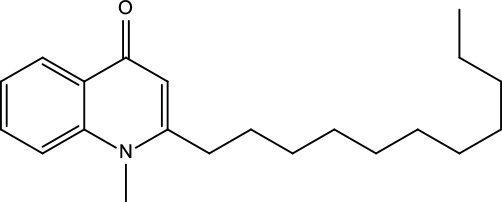	Evodiae Fructus
MOL145	1-Methyl-2-nonyl-4-quinolone	48.42	0.2	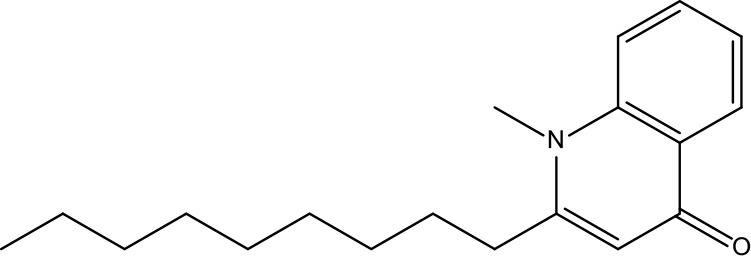	Evodiae Fructus
MOL147	Evocarpine	48.66	0.36	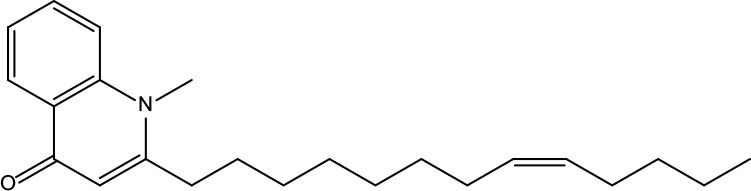	Evodiae Fructus
MOL148	Icosa-11,14,17-trienoic acid methyl ester	44.81	0.23	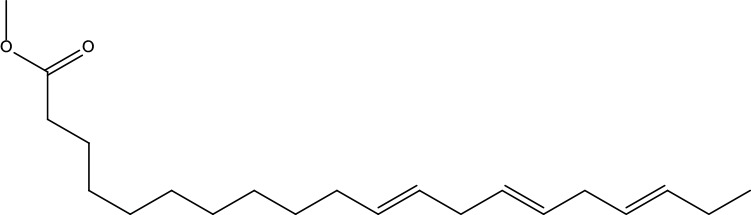	Evodiae Fructus
MOL161	2-Hydroxy-3-formyl-7-methoxycarbazole	83.08	0.18	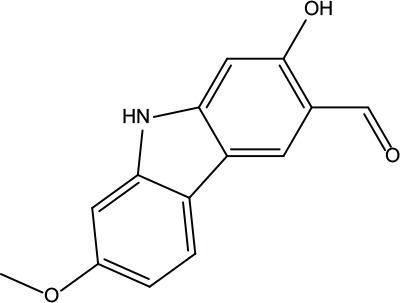	Evodiae Fructus
MOL167	24-Methyl-31-norlanost-9(11)-enol	38	0.75	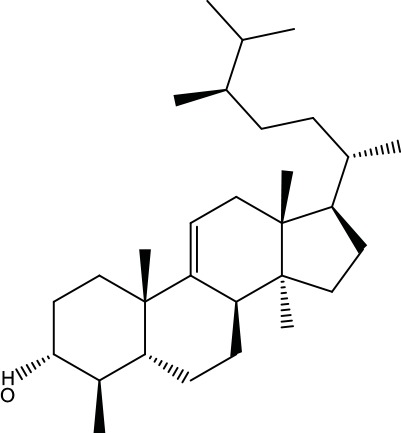	Evodiae Fructus
MOL175	5alpha-O-(3’-Methylamino-3’-phenylpropionyl)nicotaxine	30.86	0.49	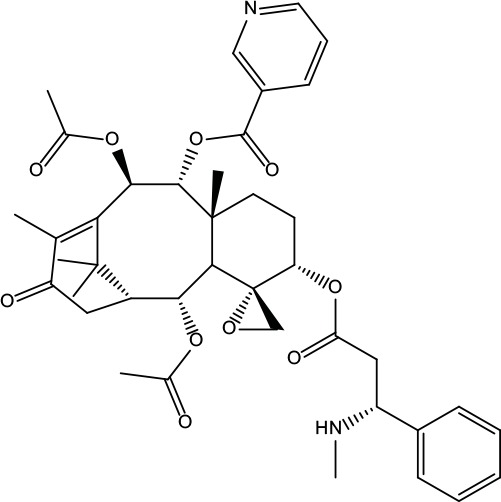	Evodiae Fructus
MOL177	6-OH-Luteolin	46.93	0.28	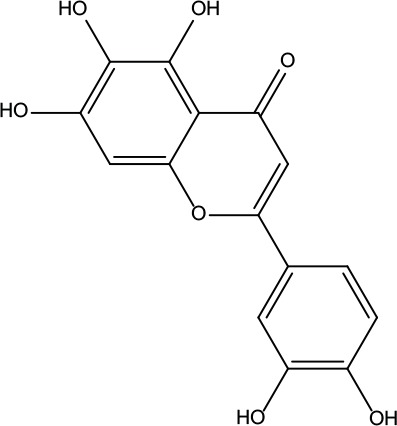	Evodiae Fructus
MOL187	Evodiamide	73.77	0.28	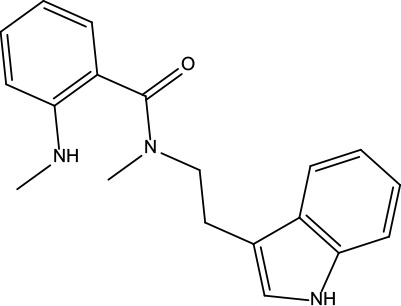	Evodiae Fructus
MOL190	Fordimine	55.11	0.26		Evodiae Fructus
MOL191	Goshuyuamide I	83.19	0.39	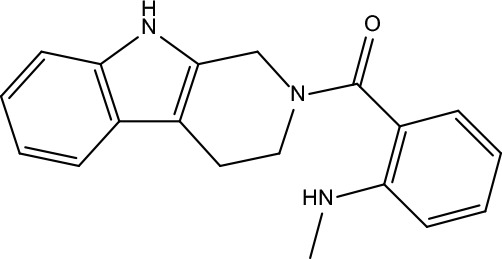	Evodiae Fructus
MOL192	GoshuyuamideII	69.11	0.43	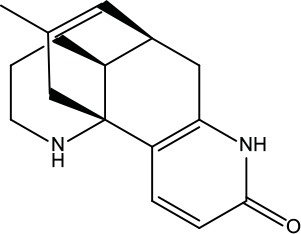	Evodiae Fructus
MOL193	Gossypetin	35	0.31	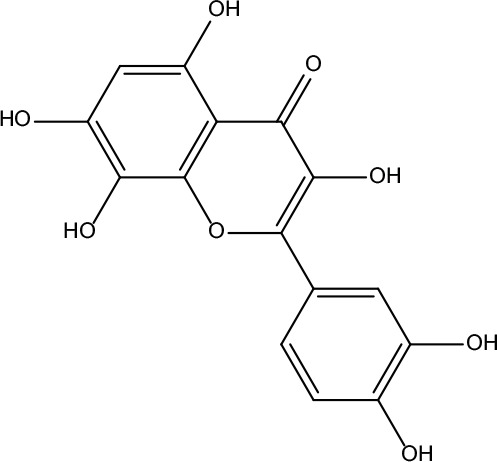	Evodiae Fructus
MOL194	Gravacridoneshlirine	63.73	0.54	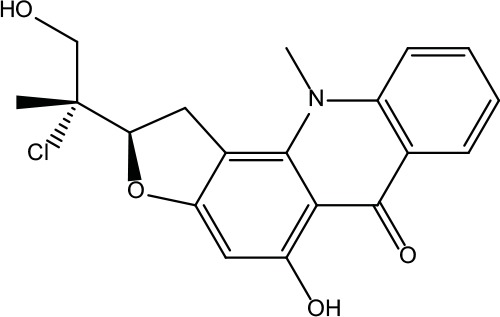	Evodiae Fructus
MOL198	N-(2-Methylaminobenzoyl)tryptamine	56.96	0.26	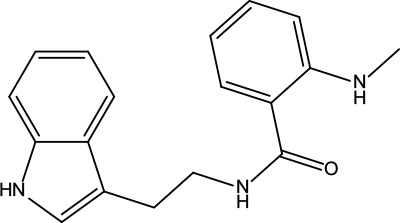	Evodiae Fructus

#### Targets Identification of ZJP on HCC

Among the 42 candidate bioactive components, 1,092 protein targets were retrieved from TCMSP database and TCMID. The detailed information was shown in **Supplement 3**. After eliminating the overlaps, 517 protein targets were obtained for further analyses. 566 HCC-related human genes were collected from OncoDB.HCC and Liverome databases. The detailed information was shown in **Supplement 4**. Then, these protein targets of ZJP were mapped with HCC using TTD, CTD, and PharmGKB. As a result, 86 targets of 32 components in ZJP were associated with HCC and the detailed information of the 86 targets of ZJP on HCC was shown in **Table 3**.

**Table 3:** Targets of ZJP on HCC.

Number	Protein name	Gene name	Score	Degree
1	Mitogen-activated protein kinase 1	MAPK1	3	52
2	Phosphatidylinositol 4,5-bisphosphate 3-kinase catalytic subunit gamma isoform	PIK3CA	7	51
3	RAF proto-oncogene serine/threonine-protein kinase	RAF1	1	42
4	Cellular tumor antigen p53	TP53	3	31
5	G1/S-specific cyclin-D1	CCND1	4	28
6	Epidermal growth factor receptor	EGFR	2	28
7	NF-kappa-B inhibitor alpha	NFKBIA	3	24
8	Cyclin-dependent kinase inhibitor 1	CDKN1A	2	21
9	Pro-epidermal growth factor	EGF	2	21
10	Myc proto-oncogene protein	MYC	2	21
11	Proto-oncogene tyrosine-protein kinase Src	SRC	1	19
12	Proto-oncogene c-Fos	FOS	2	17
13	Caspase-3	CASP3	4	16
14	Cyclin-dependent kinase 4	CDK4	1	16
15	Catenin beta-1	CTNNB1	1	14
16	Retinoblastoma-associated protein	RB1	1	14
17	Signal transducer and activator of transcription 1-alpha/beta	STAT1	1	14
18	Vascular endothelial growth factor A	VEGFA	3	14
19	Transcription factor E2F1	E2F1	1	13
20	Receptor tyrosine-protein kinase erbB-2	ERBB2	1	13
21	Focal adhesion kinase 1	PTK2	1	13
22	Apoptosis regulator BAX	BAX	4	12
23	SHC-transforming protein 1	SHC1	1	12
24	Matrix metalloproteinase-9	MMP9	4	9
25	Prostaglandin G/H synthase 2	PTGS2	26	9
26	Fibronectin	FN1	2	8
27	Intercellular adhesion molecule 1	ICAM1	2	8
28	Heat shock cognate 71 kDa protein	HSPA8	1	7
29	Protransforming growth factor alpha	TGFA	1	7
30	Stromal cell-derived factor 1	CXCL12	1	6
31	Interleukin-2	IL2	2	6
32	72 kDa type IV collagenase	MMP2	2	6
33	Ras association domain-containing protein 1	RASSF1	1	6
34	Tumor necrosis factor ligand superfamily member 10	TNFSF10	1	6
35	Cyclin-A2	CCNA2	4	5
36	Heat shock protein HSP 90	HSP90AA1	17	5
37	Tumor necrosis factor ligand superfamily member 11	TNFSF11	2	5
38	Baculoviral IAP repeat-containing protein 5	BIRC5	2	4
39	Cytochrome P450 1A2	CYP1A2	3	4
40	Cytochrome P450 2E1	CYP2E1	1	4
41	Estrogen receptor	ESR1	10	4
42	Heat shock protein beta-1	HSPB1	1	4
43	Proliferating cell nuclear antigen	PCNA	2	4
44	Urokinase-type plasminogen activator	PLAU	2	4
45	Plasminogen activator inhibitor 1	SERPINE1	1	4
46	Collagen alpha-1(I) chain	COL1A1	1	3
47	C-X-C motif chemokine 2	Cxcl2	1	3
48	Receptor tyrosine-protein kinase erbB-3	ERBB3	1	3
49	Glutathione S-transferase P	GSTP1	1	3
50	Stromelysin-1	MMP3	1	3
51	Osteopontin	SPP1	1	3
52	Androgen receptor	AR	27	2
53	Catalase	CAT	1	2
54	Claudin-4	CLDN4	1	2
55	Cytochrome P450 2B6	CYP2B6	1	2
56	Cytochrome P450 3A4	CYP3A4	3	2
57	Insulin-like growth factor-binding protein 3	IGFBP3	1	2
58	Interferon regulatory factor 1	IRF1	1	2
59	Solute carrier family 2, facilitated glucose transporter member 2	SLC2A2	1	2
60	Superoxide dismutase [Cu-Zn]	SOD1	1	2
61	Vimentin	VIM	1	2
62	Multidrug resistance protein 1	ABCB1	4	1
63	Catechol O-methyltransferase	COMT	1	1
64	Flap endonuclease 1	FEN1	1	1
65	Glycogen synthase kinase-3 alpha	GSK3A	1	1
66	78 kDa glucose-regulated protein	HSPA5	1	1
67	Insulin-like growth factor II	IGF2	1	1
68	Poly [ADP-ribose] polymerase 1	PARP1	5	1
69	Plasminogen	PLG	1	1
70	Superoxide dismutase [Mn], mitochondrial	SOD2	2	1
71	ATP-binding cassette sub-family G member 2	ABCG2	2	0
72	Apolipoprotein E	APOE	2	0
73	Carbonic anhydrase 1	CA1	1	0
74	Carbonic anhydrase 2	CA2	5	0
75	CD9 antigen	CD9	1	0
76	Sterol 26-hydroxylase, mitochondrial	CYP27A1	2	0
77	Cytochrome P450 2C8	CYP2C8	1	0
78	Cytochrome P450 2C9	CYP2C9	1	0
79	DNA excision repair protein ERCC-1	ERCC1	1	0
80	Hyaluronan synthase 2	HAS2	1	0
81	Heat shock 70 kDa protein 4	HSPA4	1	0
82	Keratin, type I cytoskeletal 19	KRT19	1	0
83	Methylated-DNA–protein-cysteine methyltransferase	MGMT	1	0
84	Metallothionein-1F	MT1F	1	0
85	DNA topoisomerase 2-alpha	TOP2A	2	0
86	DNA repair protein complementing XP-C cells	XPC	1	0

#### Compound-Target Network and Analysis

**Figure 2:**
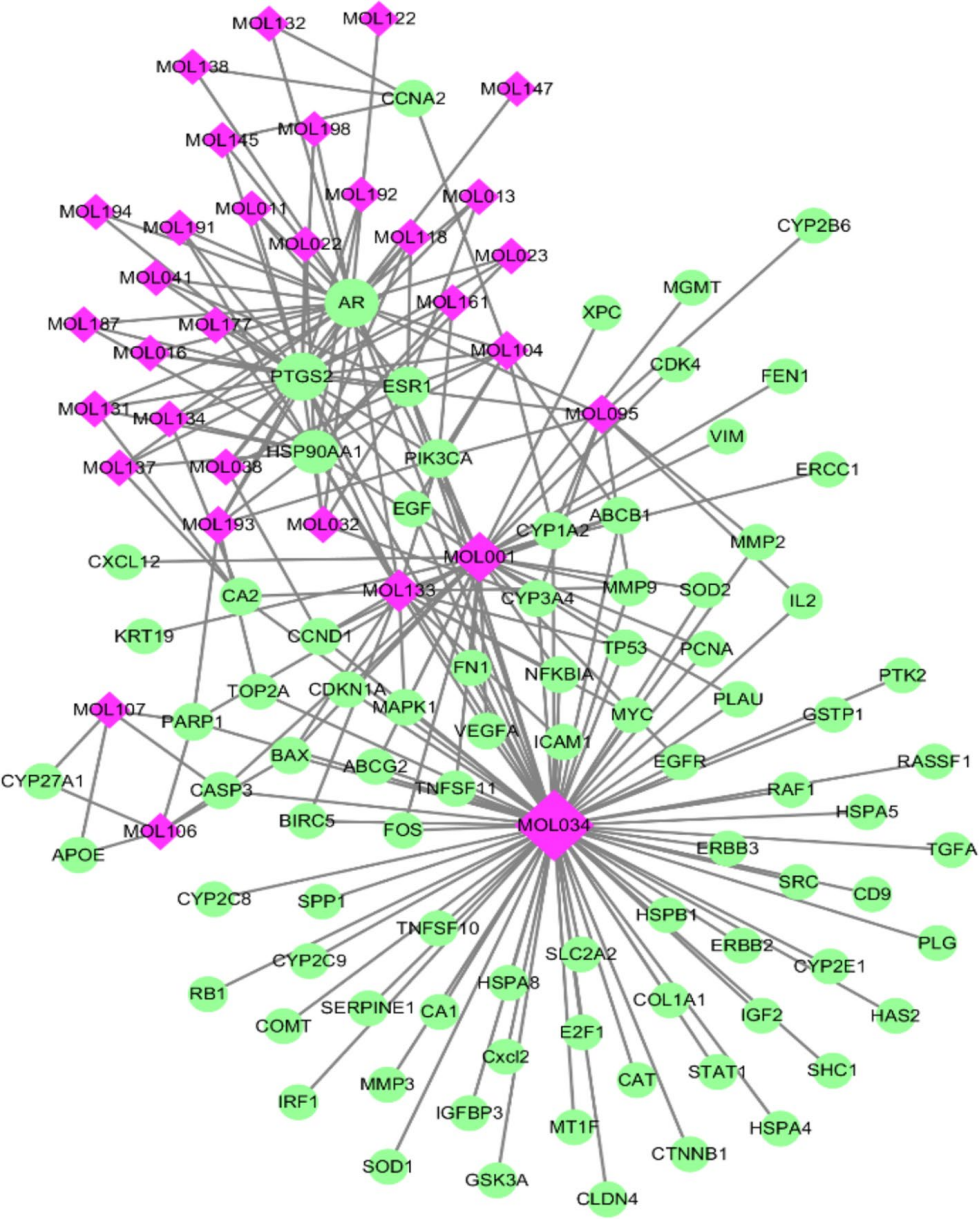
The compound-target network for ZJP on HCC. The purple nodes represent candidate active compounds and the green nodes represent potential protein targets. The edges represent the interactions between them and nodes size are proportional to their degree.

As TCM formulas exhibited multiple pharmacological activities *via* multiple targets, it was constructive for us to investigate the underlying mechanisms of TCM formulas on complex diseases by network analysis. In the current study, the compound-target network of ZJP on HCC was constructed (**Figure 2**), which included 118 nodes (32 for candidate bioactive components and 86 for potential protein targets). Among these bioactive components, there were six high-degree components associated with multiple HCC targets, namely, quercetin (MOL034, degree = 74), berberine (MOL001, degree = 31), evodiamine (MOL133, degree = 16), isorhamnetin (MOL104, degree = 9), rutaecarpine (MOL095, degree = 9), and gossypetin (MOL193, degree = 6). Among these potential protein targets, there were seven high-degree targets associated with multiple compounds, namely, AR (degree = 27), PTDG2 (degree = 26), HSP90AA1 (degree = 16), ERS1 (degree = 10), PIK3CA (degree = 7), CA2 (degree = 5), and PARP1 (degree = 5). These high-degree protein targets in the network may account for the essential therapeutic effects of ZJP on HCC.

#### GO and Pathway Enrichment Analysis

**Figure 3:**
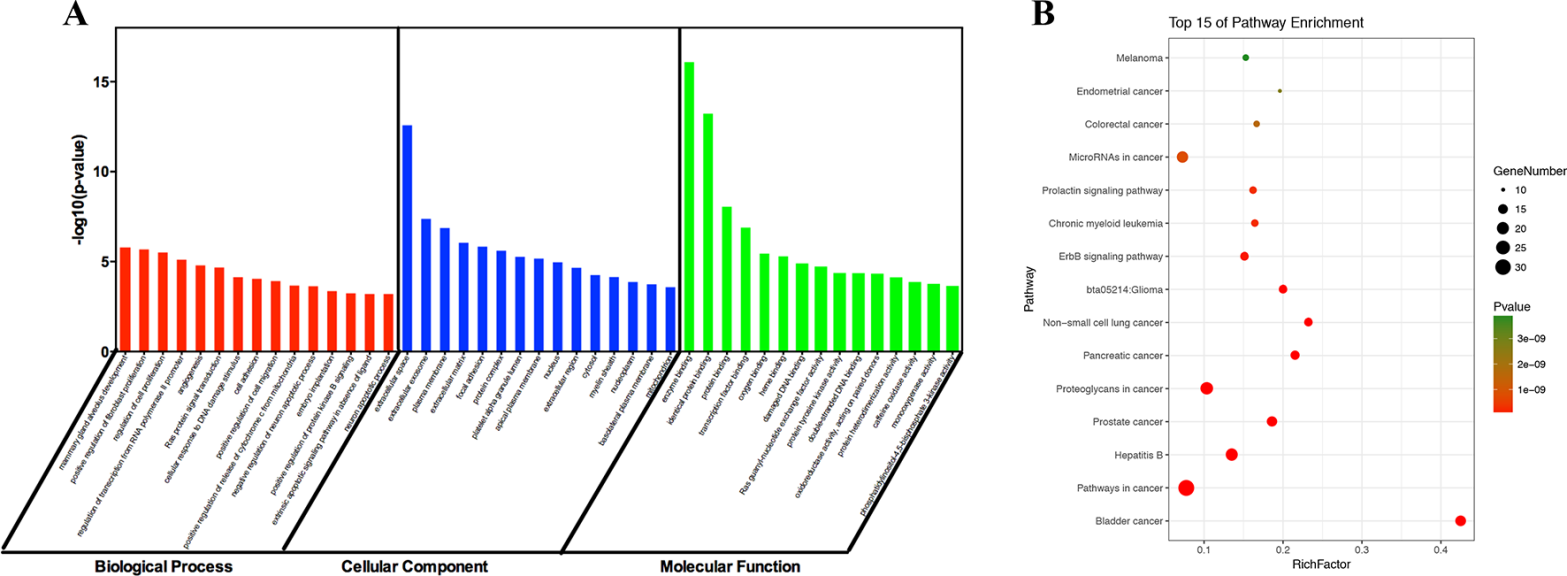
The 15 most significance of gene ontology **(A)** and pathway enrichment **(B)** analysis of therapy target genes of ZJP on HCC.

To identify the biological characteristics of putative targets of ZJP on HCC in detail, the GO and pathway enrichment analyses of involved targets were conducted *via* the functional annotation tool of DAVID Bioinformatics Resources 6.7. There were respectively 90 biological process (BP), 36 cellular component (CC), and 63 molecular function (MF) terms in total, which met the requirements of Count ≥ 2 and EASE scores ≤ 0.05. The detailed GO information was shown in **Supplement 5**. The top 15 significantly enriched terms in BP, CC, and MF categories were shown in **Figure 3A**, which indicated that ZJP may regulate cancer cell proliferation *via* enzyme binding, protein binding, and transcription factor binding in cytosol, plasma membrane, and extracellular space to exert its therapeutic effects on HCC. To explore the underlying involved pathways of ZJP on HCC, KEGG pathway analysis of involved targets was conducted. The detailed pathway information of ZJP on HCC was shown in **Supplement 6**. The top 15 significantly enriched pathways of ZJP on HCC were shown in **Figure 3B**. The pathways in cancer exhibited the largest number of involved targets (31 counts).

#### Target-Pathway Network and Analysis

**Figure 4:**
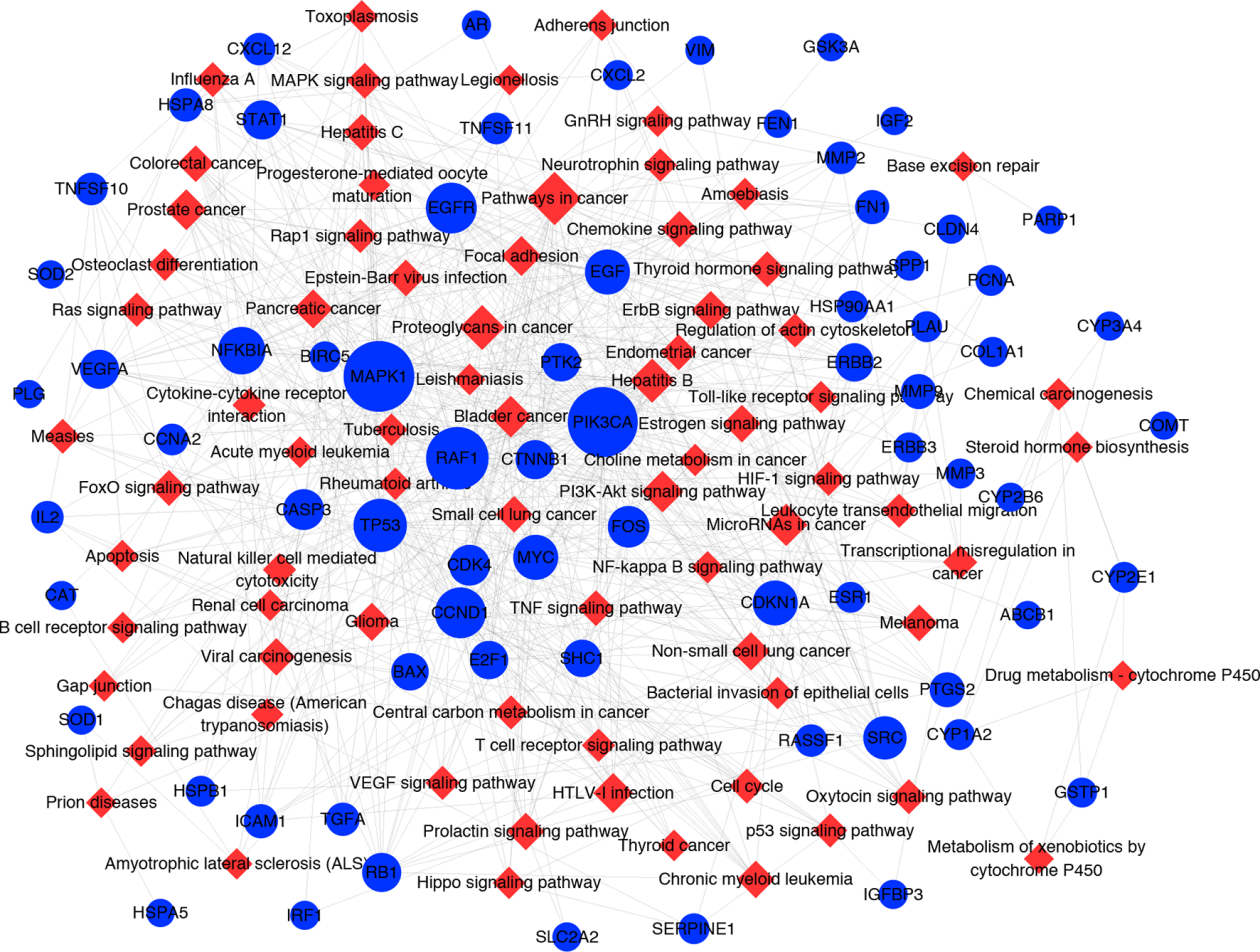
The target-pathway network for ZJP on HCC. The blue nodes represent targets and the red nodes represent pathways. The edges represent the interactions between them and node size is proportional to their degree.

It has been suggested that genes and proteins do not exhibit their biological and pharmacological activities independently. Actually, they function in interactive and dynamics pathways and networks at the cell and molecular levels (Kumar et al., 2015). To further characterize the molecular mechanism by which ZJP alleviated HCC, a target-pathway network was performed based on all involved proteins and their corresponding significant signalling pathways (**Figure 4**). This network included 145 nodes (70 for proteins and 75 for pathways). Among these potential pathways, pathway in cancer was consider the most significant one with the highest degree value. Among these potential targets, MAPK1, PIK3CA, EGFR, CCND1, and NFKBIA were identified as relatively high-degree targets, which played an essential role in cell proliferation and survival and were considered as the key markers of ZJP treatment on HCC. From the integrated drug target prediction, GO, and pathway enrichment as well as network analyses, we speculated that the antitumor effects of ZJP on HCC might be associated with the roles of its key targets including MAPK1, PIK3CA, EGFR, CCND1, and NFKBIA in regulating HCC cell proliferation and survival.

### Experimental Validation

#### ZJP-Inhibited HCC Cell Growth *in Vitro*

**Figure 5:**
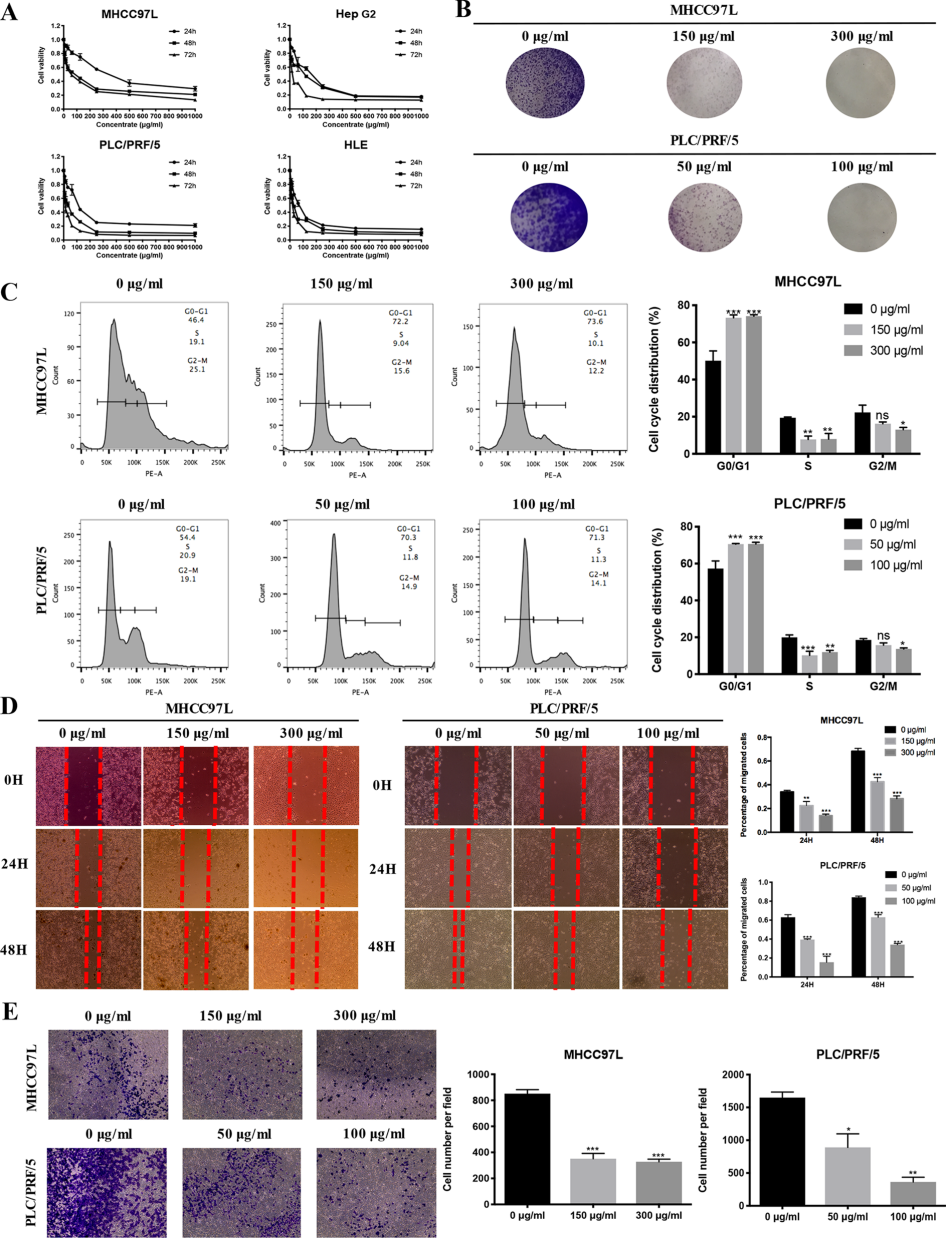
ZJP inhibited HCC cell growth *in Vitro*. **(A)** Time- and dose-dependent effects of ZJP treatment on the viability of HCC cells. **(B)** Representatives images of colony formation of MHCC97L and PLC/PRF/5 cells. **(C)** The representative images and statistical graphs of MHCC97L and PLC/PRF/5 cell cycle analysis. **(D)** The representative images and statistical graphs of migration assay of MHCC97L and PLC/PRF/5 cells. **(E)** The representative images and statistical graphs of transwell chambers of MHCC97L and PLC/PRF/5 cells. *P < 0.05, **P < 0.01, ***P < 0.001 versus the nontreated group.

To validate the antiproliferative effect of ZJP on HCC as postulated from network pharmacology analysis, various cell function assays were conducted. When exposed to increasing concentrations (7.8125-1000 µg/ml) of ZJP, a dose- and time-dependent decrease in HCC cell viability at different time points (24 h, 48 h, 72 h) was observed (**Figure 5A**). The IC50 values of ZJP in different kinds of HCC cell lines were shown in **Supplement 7**. Then, MHCC97L and PLC/PRF/5 cells were chosen for other cell function assays. As shown in **Figure 5B**, a dose-reduced colony formation was observed in MHCC97L and PLC/PRF/5 cells after treatment with increasing ZJP for 10 days. The delay of the G1/S transition in HCC cells after treatment with ZJP was further confirmed by cell cycle analysis (**Figure 5C**). Interestingly, a dose-reduced migratory and invasive property was also observed in MHCC97L and PLC/PRF/5 cells after treatment with increasing ZJP, as shown in **Figures 5D, E**. In summary, these data conformed that ZJP may inhibit HCC cell growth *in vitro* in various aspects.

#### ZJP Inhibited Tumor Growth of Orthotopic HCC Implantation Murine Model *in Vivo*

**Figure 6:**
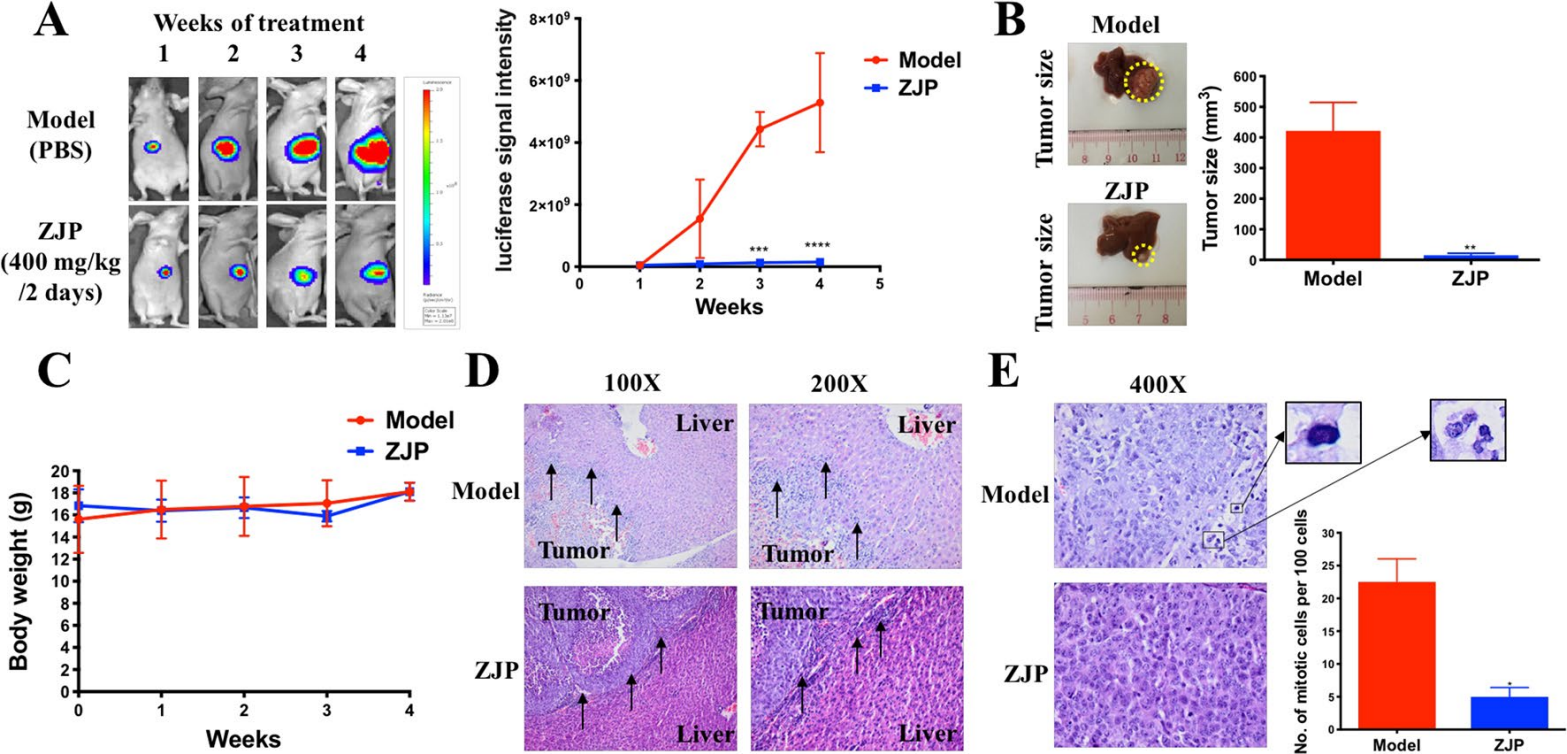
ZJP-inhibited tumor growth of orthotopic HCC implantation murine model *in vivo*. **(A)** The representative images and statistical graph of luciferase signal of animals throughout the oral treatment. **(B)** The representative images and statistical graph of tumor size at the end of experiment. **(C)** The body weight of animals throughout the experiment. **(D)** ZJP suppressed the invasion of the orthotopic tumor cells into the livers. **(E)** ZJP suppressed the mitotic events in tumors. *P < 0.05, **P < 0.01, ***P < 0.001 versus the nontreated group.

To further verify the antiproliferative effect of ZJP on HCC, an orthotopic HCC implantation murine model was established. The tumors in the model group continued to grow into large tumors, giving out very strong luminescence signals. In contrast, there was no remarkable enhancement of the luminescence signals in the ZJP-treated group during the treatment time (**Figure 6A**). By the end of the treatment course (week 4), the size of dissected tumor in the ZJP group was significantly decreased when compared with the model group (**Figure 6B**). These observations indicated that the growth rate of orthotopically implanted HCC was significantly inhibited with the treatment of ZJP. On the other hand, the model and ZJP-treated group had comparable body weight, suggesting that there was no severe toxicity in the presence of ZJP (**Figure 6C**).

Histological examination showed an irregular and invasive edge at the growth front of the tumors in the model group, revealing significant local invasion of orthotopically implanted HCC cells into the normal liver tissue (**Figure 6D**). In contrast, there was a regular and well-defined tumor growth front of the tumors in the ZJP-treated group. In addition, there was a significant inhibition of mitotic events in the ZJP-treated group when compared with the model group (**Figure 6E**), demonstrating a significant antiproliferative property of ZJP on *in vivo* growth of HCC.

#### ZJP Attenuated HCC Partially by Regulating Cell Proliferation and Cell Survival

**Figure 7:**
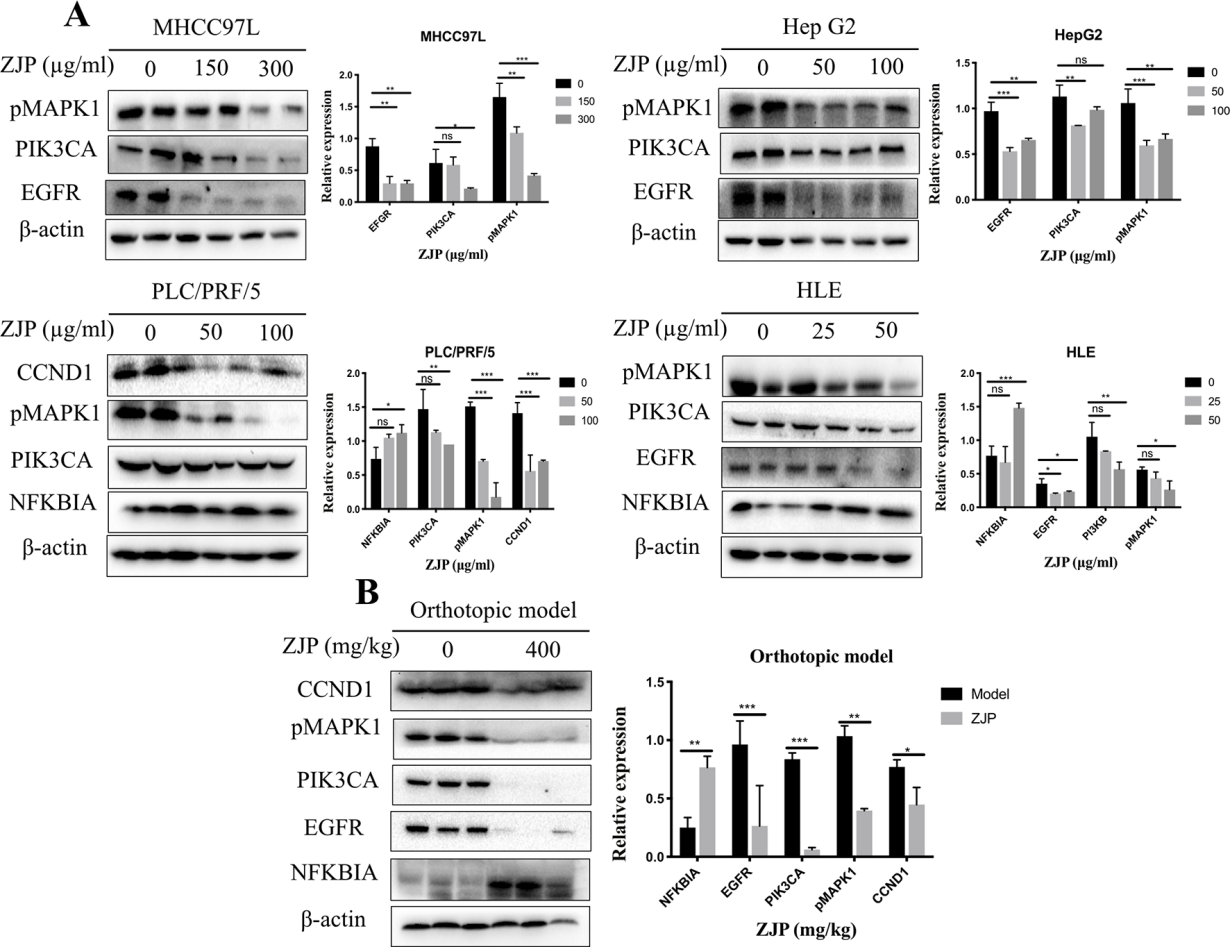
The relative expressions of related proteins with ZJP treatment on HCC cells **(A)** and orthotopic HCC implantation murine model **(B)** *P < 0.05, **P < 0.01, *** P< 0.001 versus the nontreated group.

Network pharmacology analysis predicted that the molecular targets highly associated with the common signaling pathways including MAPK1, PIK3CA, EGFR, CCND1, and NFKBIA may be related with the antitumor effects of ZJP on HCC in regulating HCC cell proliferation and survival. We further validated the antitumor properties of ZJP on the expressions of the potential targets identified *via* network pharmacology. As shown in **Figure 7A**, pretreatment of MHCC97L cells with ZJP (150 and 300 μg/ml) and Hep G2 cells with ZJP (50 and 100 μg/ml) led to apparent repression of pMAPK1, PIK3CA, and EGFR. Pretreatment of PLC/PRF/5 cells with ZJP (50 and 100 μg/ml) led to apparent enrichment of NFKBIA and repression of pMAPK1, PIK3CA, and CCND1. Likewise, pretreatment of HLE cells with ZJP (25 and 50 μg/ml) led to apparent enrichment of NFKBIA and repression of pMAPK1, PIK3CA, and EGFR. In addition, the expressions of the potential targets obtained from the tumors of orthotopic HCC implantation murine model were also examined. As shown in **Figure 7B**, the oral treatment of ZJP led to apparent enrichment of NFKBIA and repression of pMAPK1, EGFR, PIK3CA, and CCND1. These results validated that ZJP may regulate the cell proliferation and cell survival mainly through PI3K-NF-κB, EGFR-MAPK, and CCND1 pathways.

## Discussion

HCC is a complex disease with a stepwise sequence of events, which are related to multiple proteins or pathways during the development and progression (Feitelson et al., 2002). Being composed of multiple compounds, TCM may exhibit extensive pharmacological activities with multiple targets and pathways (Zhou et al., 2014), which may benefit the treatment of HCC. On the other hand, this property of TCM may bring difficulty towards the in-depth study of the underlying mechanisms. Network pharmacology approach, which integrates the systems biology and in silico technologies, may offer a direction for the mechanistic study of complicated TCM. In the current study, we used this approach to clarify the pharmacological mechanism by which ZJP alleviated HCC.

In the system of TCM, compounds lacking proper pharmacokinetical properties could not reach the target organs to deliver the biological activities. In the current study, the compounds in ZJP with OB ≥ 30% and DL index ≥ v0.18 were considered pharmacokinetically active as they are possibly absorbed and distributed in human body. In the compound-target network, compounds with high-degree may account for the major therapeutic effects of ZJP on HCC. In this study, quercetin was the most significant compound, followed by berberine, evodiamine, isorhamnetin, rutaecarpine, and gossypetin. Quercetin, a natural flavonoid, was shown an anticarcinogenic action *via* inhibiting PI3K pathway (Maurya and Vinayak, 2015). Berberine and evodiamine are the primary bioactive compound in CR and EF, respectively. The most significantly bioactive compound berberine from CR could suppress Cyclin D1 expression *via* proteasomal degradation in human hepatoma cells (Wang et al., 2016). In addition, berberine could also induce autophagic cell death and mitochondrial apoptosis in liver cancer cells (Wang et al., 2010b). The bioactive compound evodiamine from EF was shown to exert its antitumor effect on HCC *via* inducing Akt-mediated apoptosis (Yang et al., 2017). Isorhamnetin, a flavonol aglycone, obtained from the TCM *Hippophae rhamnoides L.*, was shown to exhibit *in vitro* antitumor activity against HCC cells (Teng et al., 2006). Although there was no literature relating rutaecarpine with HCC, it was reported to ameliorate the hyperlipidemia and hyperglycemia, which were the risk factors of HCC (Nie et al., 2016). Gossypetin, a naturally occurring hexahydroxy flavone, has been shown to exert anticancer potential *via* inducing apoptotic and autophagic cell death (Lee et al., 2017). All these literatures together with our experimental studies supported the conclusion of network prediction and demonstrated a successful practice of network pharmacology approach in identification of action mechanism of TCM.

**Figure 8:**
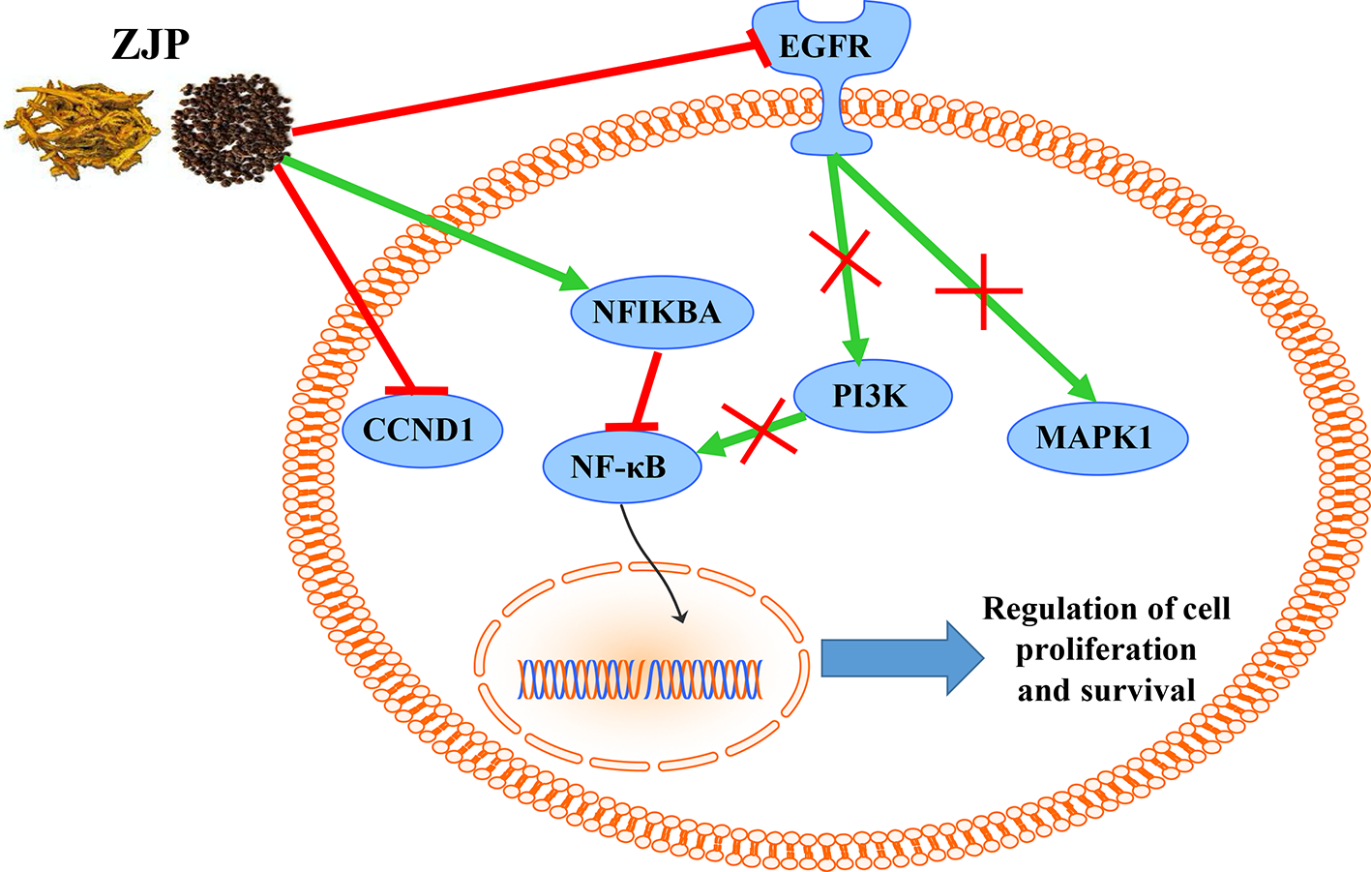
The overall regulatory network involved in the inhibitory effect of ZJP on HCC.

From the integrated drug target prediction and pathway analysis, ZJP may exert its antitumor effects on HCC *via* the regulation of cell proliferation and survival, which was characterized as the important mechanism of liver cancer progression (Llovet and Bruix, 2008). Signaling pathways that control multiple processes, such as cell proliferation, invasion, metastasis, and angiogenesis, are commonly dysregulated in the pathological progression of HCC, which have become an important source of targets from a therapeutic perspective in HCC treatment (Moeini et al., 2012). As predicted by network pharmacology approach, ZJP may exert therapeutic effects on HCC primarily by regulating HCC cell proliferation and cell survival *via* PI3K-NF-κB, EGFR-MAPK, and CCND1 signaling pathways. To further validate the postulation, we investigated the curative effects of ZJP on different kinds of HCC cells *in vitro* and orthotopic HCC implantation murine model *in vivo*. The *in vitro* results showed that ZJP treatment significantly decreased HCC cell viability and colony formation ability, halted the cells in GO/G1 phase, and inhibited the cell migration and invasion activities in a dose-dependent manner. The *in vivo* results revealed that oral treatment of ZJP significantly decreased tumor growth and invasion. Especially, the expression levels of PIK3CA, EGFR, pMAPK1, and CCND1 were significantly decreased, whereas NFKBIA was significantly increased. NFKBIA is a member of the NF-κB inhibitor family. It could inhibit NF-κB, which has an essential role in promoting cell survival *via* immune, inflammatory, and stress responses. The aberrant regulation of NF-κB and the signaling pathways that control its activity are related to cancer development and progression (Baud and Karin, 2009). PIK3CA has been shown to be mutated in multiple tumors and considered as a potential therapeutic target (Hanker et al., 2013). The PI3K signaling pathway has an essential role in regulating the proliferation, apoptosis, and metastasis of tumor cells, which could be hyperactivated *via* activating mutations in PIK3CA. It is a key juncture in signaling communication network and it makes crosstalk between several important pathways, including NF-κB (Engelman, 2009). EGFR is a transmembrane glycoprotein, which is a member of the protein kinase superfamily. As a receptor for members of the epidermal growth factor family, binding this protein with ligand would trigger tyrosine kinase activity and lead to cell proliferation. EGFR was usually overexpressed at both mRNA and protein levels in HCC (Villanueva et al., 2008). Specifically, the EGFR signaling pathway has a pivotal role in regulating the multiple processes of HCC, such as cell proliferation, angiogenesis, invasion, and metastasis (Wang et al., 2015b). Therefore, when HCC cells were pretreated with ZJP, the decreased expression of EGFR may partially contribute to its curative effects. MAPK1 is one of the members of the MAP kinase family. MAP kinases, also known as extracellular signal-regulated kinases, are associated with a wide variety of biochemical processes, including proliferation and differentiation. In HCC, the activation of MAPK pathway might result from aberrant upstream signals, such as aberrant EGFR signaling (Calvisi et al., 2006). After phosphorylation, it could translocate to the nucleus and phosphorylate the nuclear targets. From the experimental validation, effective blockade of EGFR and pMAPK1 in HCC cells after pretreatment with ZJP may contribute to its curative effects. Cyclin D1 (CCND1) is a member of D-type cyclin family, which also includes cyclin D2 and D3. CCND1 could promote the transition from the G1 to S phase of the cell cycle (Fu et al., 2004). It has been shown that overexpression of CCND1 in liver can induce HCC in a transgenic mice model (Deane et al., 2001). Taken together, our findings suggested that ZJP may inhibit HCC progression mainly *via* the regulation of cell proliferation and survival through the EGFR-MAPK, PI3K-NF-κB, and CCND1 signaling pathways (**Figure 8**). Furthermore detailed pharmacological mechanisms by which ZJP ameliorates HCC will be investigated in our future study.

## Conclusion

In conclusion, the pharmacological mechanism by which ZJP inhibited HCC was investigated with the combination of network pharmacology prediction and experimental validation. We demonstrated that ZJP may inhibit the proliferation and survival of HCC mainly *via* the regulation of EGFR/MAPK, PI3K/NF-κB, and CCND1 signaling pathways. Our study further suggested combined network pharmacology prediction and experimental validation study may offer a useful tool to characterize the action mechanism of TCM in detail. The potential therapeutic effects of ZJP on HCC may benefit from the further studies on clinical trials of HCC patients with ZJP treatment.

## Data Availability Statement

The raw data supporting the conclusions of this manuscript will be made available by the authors, without undue reservation, to any qualified researcher.

## Ethics Statement

All animals received human care throughout the experiments and the study protocols were approved by the Committee on the Use of Live Animals in Teaching and Research (CULATR).

## Author Contributions

YF designed the experiments, analyzed the data, and prepared the manuscript. WG and JH conducted the experiments, analyzed data, and prepared the manuscript. NW and H-YT conducted the experiments, and FC and FYC revised the manuscript. All authors confirmed the final manuscript.

## Funding

This study was supported by the Research Grant Council, the HKSAR (Project code: RGC GRF 17152116), the Commissioner for Innovation Technology, the HKSAR (Project code: ITS/091/16FX), and the Health and Medical Research Fund (HMRF) (Project code: 16172751).

## Conflict of Interest

The authors declare that the research was conducted in the absence of any commercial or financial relationships that could be construed as a potential conflict of interest.

## Abbreviations

ATCC, American type culture collection; ADME, absorption, distribution, metabolism, and excretion; BP, biological process; BSA, bovine serum albumin; CC, cellular component; CCND1, cyclin D1; CR, coptidis Rhizoma; CTD, comparative toxicogenomics database; CULATR, committee on the use of live animals in teaching and research; DL, drug-likeness; EASE, expression analysis systematic explorer; EF, evodiae fructus; GO, gene ontology; HCC, hepatocellular carcinoma; MF, molecular function; OB, oral bioavailability; PVDF, polyvinylidene fluoride; SDS-PAGE, sodium dodecyl sulfate-polyacrylamide gel electrophoresis; TBST, tris buffered saline-tween 20; TCM, traditional Chinese medicine; TCMID, traditional Chinese medicine integrated database; TCMSP, traditional Chinese medicine systems pharmacology; TTD, therapeutic target database; ZJP, Zuojin pill.

## Supplementary Material

The Supplementary Material for this article can be found at: https://www.frontiersin.org/articles/10.3389/fphar.2019.01185/full#supplementary-material

Supplement 1 The chromatographic fingerprinting of ZJP and standards.

Supplement 2 The summary of total components in ZJP.

Supplement 3 The summary of protein targets from the candidate bioactive components in ZJP.

Supplement 4 The detailed information of 566 HCC-related human genes.

Supplement 5 The detailed GO information of terms which met the requirements of Count ≥2 and EASE scores ≤0.05.

Supplement 6 The detailed pathway information of ZJP on HCC.

Supplement 7 The IC50 of ZJP in different kinds of HCC cell lines.
