# Supplementary material for: Integrating Network Pharmacology and Pharmacological Evaluation for Deciphering the Action Mechanism of Herbal Formula Zuojin Pill in Suppressing Hepatocellular Carcinoma
Source: Front Pharmacol. 2019 Oct 9;10:1185. doi: 10.3389/fphar.2019.01185 (PMC6795061; doi:10.3389/fphar.2019.01185)
Supplement: Supplement 1 — The chromatographic fingerprinting of ZJP and standards. [file DataSheet_1.zip › Supplements/Supplement 7.docx]

**Supplement 7. The IC50 of ZJP in different kinds of HCC cell lines.**

| IC50 (µg/ml) | 24h | 48h | 72h |
| --- | --- | --- | --- |
| MHCC97L | 339.3 | 73.22 | 51.96 |
| Hep G2 | 135.8 | 77.45 | 20.43 |
| PLC/PRF/5 | 116.2 | 25.99 | 12.23 |
| HLE | 66.06 | 28.16 | 17.98 |
