# Supplementary figures and images for: Integrating Network Pharmacology and Pharmacological Evaluation for Deciphering the Action Mechanism of Herbal Formula Zuojin Pill in Suppressing Hepatocellular Carcinoma
Source: Front Pharmacol. 2019 Oct 9;10:1185. doi: 10.3389/fphar.2019.01185 (PMC6795061; doi:10.3389/fphar.2019.01185)

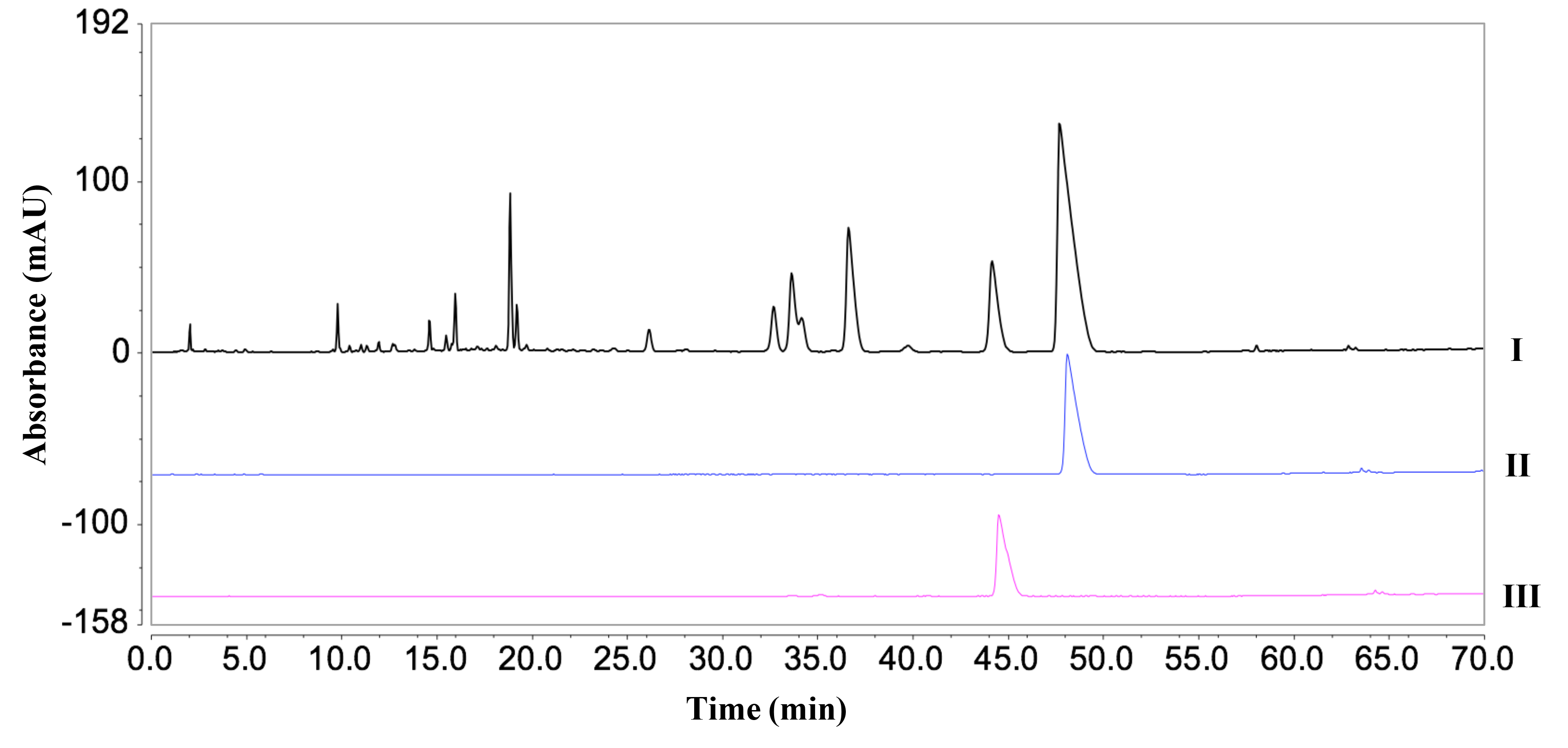

Supplement: Supplement 1 — The chromatographic fingerprinting of ZJP and standards. [file DataSheet_1.zip › Supplements/Supplement 1.tif]
